# Differences between pathologic and non-pathologic high myopia in 4-year outcomes of anti-VEGF therapy for macular neovascularization

**DOI:** 10.1038/s41598-024-64456-z

**Published:** 2024-06-11

**Authors:** Yuki Honda, Manabu Miyata, Masahiro Miyake, Masayuki Hata, Shogo Numa, Yuki Mori, Sotaro Ooto, Hiroshi Tamura, Naoko Ueda-Arakawa, Yuki Muraoka, Ayako Takahashi, Keina Sado, Ai Kido, Akitaka Tsujikawa

**Affiliations:** Department of Ophthalmology and Visual Sciences, Kyoto University Graduate School of Medicine, 54 Shogoin-kawahara-cho, Sakyo-ku, Kyoto City, Kyoto Prefecture 606-8507 Japan

**Keywords:** Aflibercept, Chorioretinal atrophy, Macular neovascularization, META-PM study, Pathologic myopia, Ranibizumab, Diseases, Medical research

## Abstract

This retrospective observational study aimed to investigate the difference in 4-year outcomes of ranibizumab or aflibercept therapy for macular neovascularization (MNV) with high myopia between pathologic myopia (PM) and non-PM. This study was conducted at Kyoto University Hospital and included consecutive treatment-naïve eyes with active myopic MNV, in which a single intravitreal ranibizumab or aflibercept injection was administered, followed by a pro re nata (PRN) regimen for 4 years. Based on the META-PM study classification, eyes were assigned to the non-PM and PM groups. This study analyzed 118 eyes of 118 patients (non-PM group, 19 eyes; PM group, 99 eyes). Baseline, 1-year, and 2-year best-corrected visual acuity (BCVA) were significantly better in the non-PM group (*P* = 0.02, 0.01, and 0.02, respectively); however, the 3-year and 4-year BCVA were not. The 4-year BCVA course was similar in both groups. However, the total number of injections over 4 years was significantly higher in the non-PM than in the PM group (4.6 ± 2.6 vs. 2.9 ± 2.6, *P* = 0.001). Four-year BCVA significantly correlated only with baseline BCVA in both non-PM (*P* = 0.047, β = 0.46) and PM groups (*P* < 0.001, β = 0.59). In conclusion, over the 4-year observation period, the BCVA course after anti-VEGF therapy for myopic MNV was similar in the eyes with non-PM and those with PM; however, more additional injections in a PRN regimen were required in the eyes with non-PM compared to those with PM. Thus, more frequent and careful follow-up is required for the eyes with non-PM compared with those with PM to maintain long-term BCVA.

## Introduction

High myopia, which potentially threatens vision, is globally increasing toward the future and become a social problem^1^. In particular, macular neovascularization (MNV) in high myopia has an extremely inferior natural course as best-corrected visual acuity (BCVA) decreased to 20/200 or less in 96.3% of the eyes over 10 years, reported previously^2^. Another recent 10-year observational study showed that intravitreal ranibizumab injection (IVR) and/or intravitreal aflibercept injection (IVA) was effective and safe for the eyes with MNV and pathologic myopia (PM) because none of the studied 26 eyes had BCVA decreased of 20/200 or less or drug-induced complications^3^.

In 2015, the META-PM study group proposed an international photographic classification and grading system for myopic maculopathy^4^, which has been widely used in various studies^3,5–9^. This study defined category 2 or higher as PM and MNV as one of the “plus” lesions that could affect central vision. Previous studies examining MNV in PM showed the outcomes of anti-VEGF therapy^3,10,11^; whereas, to the best of our knowledge, only one previous study focused on high myopia without PM^5^, demonstrating a difference in the outcomes of anti-VEGF therapy between non-PM and PM during a 2-year observation period. However, no prior study on this topic has been conducted for more than 2 years. The mean ages of patients who first received anti-VEGF therapy for MNV in high myopia and neovascular age-related macular degeneration (nAMD) were 62.8 ± 8.9 and 73.2 ± 7.4 years, respectively^3,12^. Considering the longer life expectancy in patients with myopic MNV, longer-term observation studies are desired.

Here, we investigated the difference in 4-year outcomes of anti-VEGF therapy for MNV with high myopia between non-PM and PM. Furthermore, from this perspective, we sought points to be cared for in the daily clinical practice to maintain long-term BCVA in eyes with highly myopic MNV.

## Methods

The retrospective observational study was conducted at Kyoto University Hospital (Kyoto, Japan) and was approved by the ethics committee of Kyoto University Graduate School of Medicine (Kyoto, Japan). All study protocols adhered to the tenets of the Declaration of Helsinki. We explained the nature of the study and the possible risks and benefits of participation to all study candidates who agreed to participate and provided written informed consent.

### Participants

This retrospective observational study investigated consecutive highly myopic eyes with MNV. The inclusion criteria were (1) treatment-naïve eyes with active myopic MNV diagnosed by multiple retinal specialists; (2) a single injection of 0.5 mg IVR (Lucentis, Novartis, Basel, Switzerland) or 2 mg IVA (Eylea, Bayer, Leverkusen, Germany) administered at Kyoto University Hospital between April 2009 and December 2017, followed by a pro re nata (PRN) regimen of IVR and/or IVA with follow-up for 4 years; and (3) axial length (AL) ≥ 26.00 mm. The exclusion criteria were (1) previous history of ocular surgery except for cataract surgery; (2) other macular abnormalities at baseline, including macular hole, retinal vein occlusion, diabetic retinopathy, and advanced glaucoma; and (3) no available optical coherence tomography (OCT) or infrared/color fundus images at baseline or 4 years after the initial injection. When both eyes met the above criteria, the first-treated eye was selected for analysis. Thus, one eye per patient was enrolled.

### Ophthalmological examination

Prior to the initial anti-VEGF therapy (IVR or IVA), retinal specialists diagnosed active MNV based on a comprehensive ophthalmological examination, including autorefractometry, BCVA measurements using a Landolt chart, measurement of intraocular pressure, measurement of AL using partial coherence interferometry, indirect ophthalmoscopy, slit-lamp biomicroscopy, color fundus photography, OCT, and fundus fluorescein and indocyanine green angiography (FA and ICGA, respectively). After the initial anti-VEGF therapy, all eyes were followed up essentially by measurement of BCVA and intraocular pressure, slit-lamp biomicroscopy, indirect ophthalmoscopy, color fundus photography, and OCT. Based on these follow-up examinations, retinal specialists determined the necessity of additional IVR or IVA (PRN regimen). Our policy was to perform additional treatments intensively, even for small exudates.

### Morphological analysis

At baseline (before the initial treatment), central retinal thickness (CRT) was measured as the distance between the inner border of the internal limiting membrane and the inner border of the retinal pigment epithelium, and central choroidal thickness (CCT) was measured as the distance between the outer surface of Bruch’s membrane and the chorioscleral interface on OCT B-scan images through the fovea using a built-in OCT software by one investigator (YH). The MNV area was manually measured in early-phase FA images using built-in software because the correlation between 2-year BCVA after intravitreal bevacizumab injection and MNV size was demonstrated in a previous study^13^. Since 2-year BCVA was reported to be better in juxtafoveal MNV than in subfoveal MNV^14^, the MNV location was also investigated using FA, ICGA, and OCT as follows: MNV location involving the fovea was defined as subfoveal, 0 < fovea–MNV location distance < 200 μm was defined as juxtafoveal, and fovea–MNV location distance ≥ 200 μm far from the fovea was defined as extrafoveal. Baseline and 4-year epiretinal membrane (ERM), baseline and 4-year lamellar macular hole, and baseline MNV type (type 1 or 2) were investigated using OCT images by one investigator (YH). Baseline and 4-year lens status (phakia, pseudophakia, or aphakia), cataract surgery during the 4-year study period, and the number of injections and type of anti-VEGF agent were identified based on medical records.

### Group assignment

**Figure 1:**
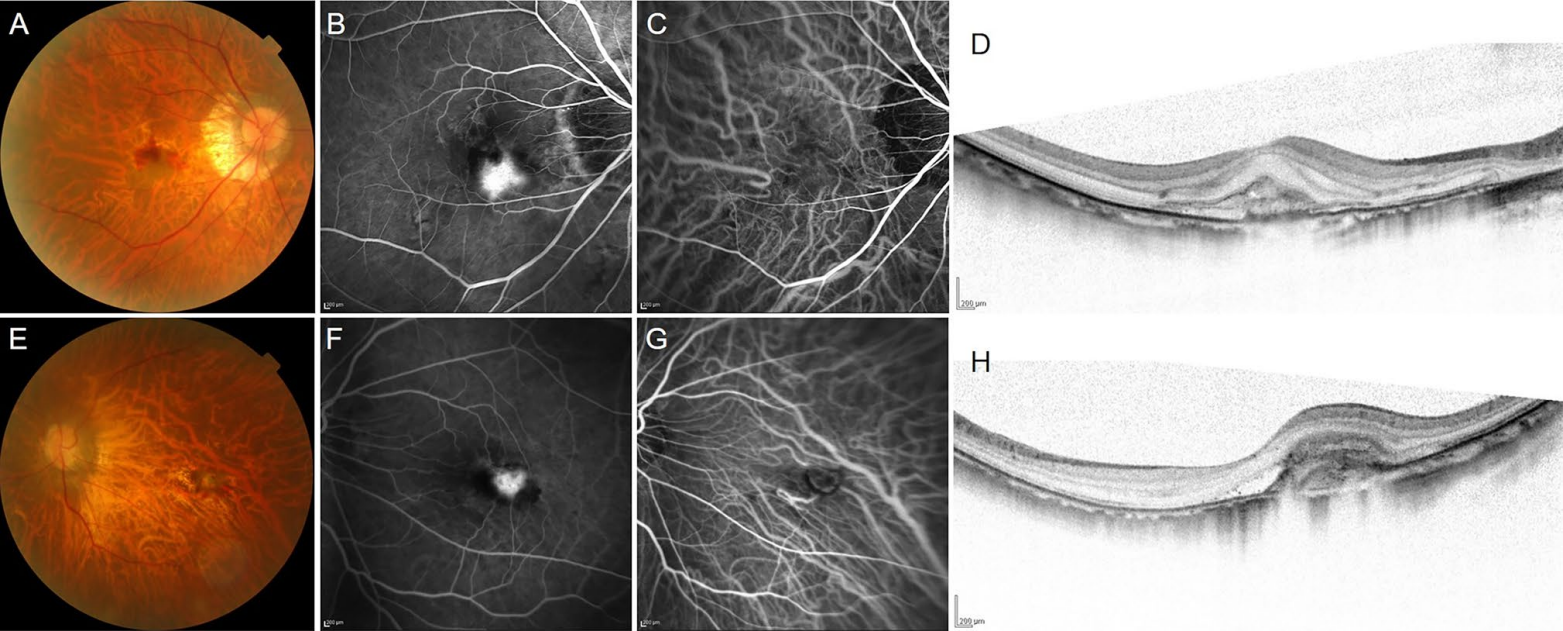
Representative images of the eyes in the non-PM and PM groups before treatment. (**A**–**D**) Images of the right eye of a woman in her 60 s in the non-PM group. The baseline and 4-year best-corrected visual acuity (BCVA) were 20/200 and 20/40, respectively. The axial length (AL) was 28.87 mm. Eight intravitreal anti-VEGF injections were required in 4 years. (**A**) A color fundus photograph. Gray-colored macular neovascularization (MNV) and subretinal hemorrhage (SRH) are also observed. The META-PM study category was determined as 1. (**B**, **C**) Images of fluorescein and indocyanine green fundus angiography at 3 min 12 s. The leakage from MNV can be observed. (**D**) A horizontal B-scan image of spectral-domain optical coherence tomography (SD-OCT). Type 2 MNV and SRH can be observed. (**E**–**H**) Images of the left eye of a woman in her 50 s in the PM group. The baseline and 4-year BCVA were 20/200 at both time points. The AL was 29.80 mm. One initial intravitreal anti-VEGF injection and no additional injection were required in 4 years. (**E**) A color fundus photograph. Gray-colored MNV and SRH are observed. The META-PM study category was determined as 2. (**F**, **G**) Images of fluorescein and indocyanine green fundus angiography at 3 min 15 s. The leakage from MNV can be observed. (**H**) A horizontal B-scan image of SD-OCT. Type 2 MNV, SRH, and subretinal fluid can be observed.

The META-PM study category at baseline was determined using color fundus photographs and referring to OCT images (Fig. 1)^3,4^. Briefly, category 0 was defined as no macular lesion, category 1 was defined as only tessellated fundus, category 2 was defined as diffuse chorioretinal atrophy, category 3 was defined as patchy chorioretinal atrophy, and category 4 was defined as macular atrophy. Two investigators (YH and KS) independently assessed each image and determined the category. When the results of the two investigators differed, a third retinal specialist (M. Miyata) provided a final judgment. Since pathologic myopia was defined as being equal to or more severe than category 2^4^, we assigned the eyes with category 0 or 1 to the non-PM group and those with category 2, 3, or 4 to the PM group. Similarly, we assessed the eyes in the non-PM group 4 years after the initial treatment.

### Statistical analysis

Data are presented as mean ± standard deviation, where applicable. For statistical analysis, BCVA was converted into the logarithm of the minimum angle of resolution (logMAR) values. To investigate inter-group differences in the number of each agent, we calculated the ratio of IVR against the total number of injections. Comparative analyses of data between the two unpaired groups were performed using the Mann–Whitney U test or chi-square test, where applicable. Changes in BCVA over 4 years were analyzed using repeated measures analysis of variance (ANOVA) and post hoc analysis with Bonferroni correction. Univariable correlation analyses between 4-year logMAR BCVA and baseline or treatment-associated parameters were performed using Spearman’s correlation test. Multivariable correlation analyses were performed using the 4-year logMAR BCVA as the dependent variable and the parameters with *P*-values < 0.10 in Spearman’s correlation test as independent variables. A *P*-value < 0.05 was considered statistically significant. All statistical analyses were conducted using SPSS version 27 software (IBM Corp., Armonk, NY, USA).

## Results

We analyzed 118 eyes of 118 consecutive patients who met the eligibility criteria. The age was 66.4 ± 9.8 (range, 38–85) years, and 91 (77%) participants were women. The AL was 29.34 ± 1.66 mm. The total number of anti-VEGF injections was 3.1 ± 2.7, of which 1.9 ± 2.1 were IVR and 1.2 ± 2.3 were IVA. Among the 118 enrolled eyes, 19 and 99 were assigned to the non-PM and PM groups, respectively, based on the META-PM study categories (Table 1). Comparing the characteristics between the two groups, the AL was significantly lower in the non-PM group than in the PM group (28.53 ± 0.94 vs. 29.49 ± 1.73, *P* = 0.03). Additionally, the proportion of men was significantly lower in the non-PM group (5% [1/19] vs. 26% [26/99], *P* = 0.046).

**Table 1:** Differences in the studied parameters between the non-PM and PM groups.

		Non-PM group	PM group	*P*-value
Eyes, n		19	99	
Age at baseline, years		63.4 ± 9.6	66.9 ± 9.8	0.09^†^
AL at baseline, mm		28.53 ± 0.94	29.49 ± 1.73	0.03*
Male sex, n (%)		1 (5)	26 (26)	0.046^†^*
LogMAR BCVA	Baseline	0.29 ± 0.34	0.52 ± 0.39	0.02*
	1-year	0.12 ± 0.22	0.37 ± 0.40	0.01*
	2-year	0.20 ± 0.33	0.40 ± 0.43	0.02*
	3-year	0.28 ± 0.38	0.41 ± 0.42	0.10
	4-year	0.28 ± 0.36	0.46 ± 0.41	0.07
MNV location at baseline (extrafoveal/juxtafoveal/subfoveal), n		6/0/13	23/20/56	0.67^‡^
MNV size at baseline, mm^2^		0.42 ± 0.49	0.57 ± 0.65	0.52
CRT at baseline, μm		288.9 ± 107.3	291.1 ± 153.5	0.82
CCT at baseline, μm		74.2 ± 36.7	57.9 ± 22.3	0.06
Number of injections, n	1st year (0–1 year)	2. 9 ± 1.3	1.9 ± 1.2	0.002*
	2nd year (1–2 years)	0.9 ± 1.1	0.5 ± 1.0	0.02*
	3rd year (2–3 years)	0.6 ± 1.0	0.2 ± 0.7	0.002*
	4th year (3–4 years)	0.2 ± 0.5	0.2 ± 0.6	0.97
	Total (0–4 years)	4.6 ± 2.6	2.9 ± 2.6	0.001*
Ratio of IVR against the total number of injections		0.70 ± 0.45	0.59 ± 0.45	0.18
No additional treatment, n (%)		3 (16)	35 (35)	0.095^†^
ERM, n (%)	Baseline	2 (11)	20 (20)	0.32^†^
	4-year	3 (16)	31 (31)	0.17^†^
LMH, n (%)	Baseline	0 (0)	0 (0)	–
	4-year	0 (0)	3 (3)	0.44^†^
Lens status, n (phakia/pseudophakia/aphakia)	Baseline	14/5/0	67/31/1	0.82^‡^
	4-year	11/8/0	43/55/1	0.48^‡^

Data are presented as means ± standard deviations where applicable.

PM = pathologic myopia; logMAR BCVA = logarithm of the minimal angle of resolution best-corrected visual acuity; AL = axial length; MNV = macular neovascularization; CRT = central retinal thickness; CCT = central choroidal thickness; IVR = intravitreal injection of ranibizumab; ERM = epiretinal membrane; LMH = lamellar macular hole.

*, Statistically significant (*P* < 0.05).

^†^, Chi-square test; ^‡^, chi-square trend test; for the others, the Mann–Whitney U test was used.

### Best-corrected visual acuity

**Figure 2:**
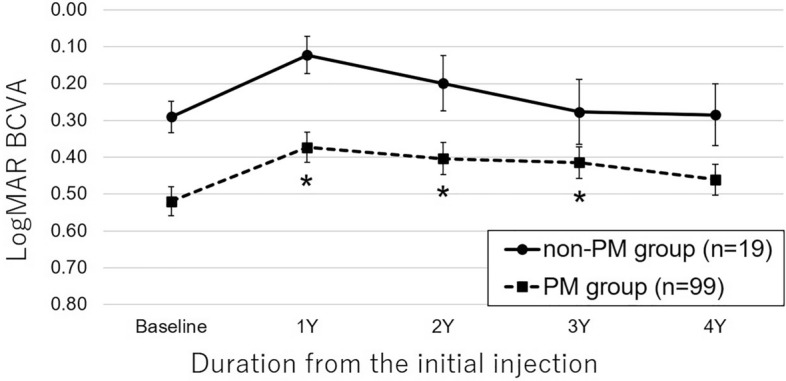
Four-year course of logarithm of the minimum angle of resolution (logMAR) best-corrected visual acuity (BCVA) values in the non-PM and PM groups. The 4-year course of logMAR BCVA values were similar for the non-PM (solid line) and PM (dashed line) groups. The 4-year logMAR BCVA values were not significantly different from the baseline values in both groups. In the PM group, the 1-, 2-, and 3-year logMAR BCVA values were significantly improved (*) compared to the baseline value. The vertical bars represent standard errors.

Baseline, 1-year, and 2-year logMAR BCVA values were better in the non-PM group (*P* = 0.02, 0.01, and 0.02, respectively); however, the 3-year and 4-year BCVA values did not significantly differ between the two study groups. The 4-year course of logMAR BCVA in both groups was similar (Fig. 2). Repeated measures ANOVA revealed that the 4-year logMAR BCVA was not significantly different from the baseline logMAR BCVA in both groups. In the PM group, the 1-, 2-, and 3-year logMAR BCVA values were significantly improved compared to the baseline value (*P* < 0.001, *P* = 0.01, and *P* = 0.02, respectively).

### Number of injection

**Figure 3:**
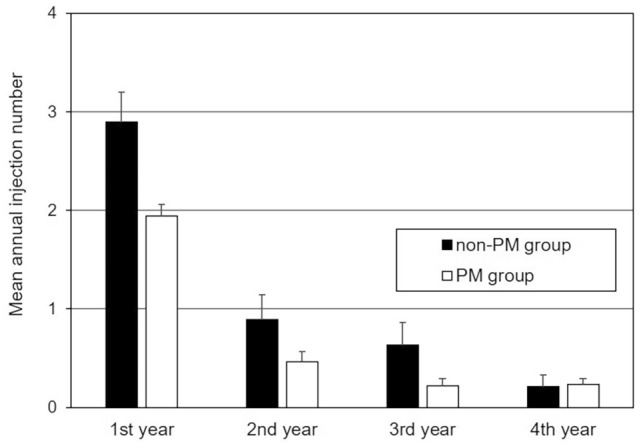
Four-year course of annual number of injections. In both the non-PM and PM groups, the annual number of injections decreased, and the levels of almost no additional injections (mean number of injections, 0.2) were reached in the non-PM group in the 4th and in the PM group in the 3rd year. The vertical bars represent positive standard errors.

The total number of injections over 4 years was significantly higher in the non-PM than in the PM group (4.6 ± 2.6 vs. 2.9 ± 2.6, *P* = 0.001). No additional injections were required in 3 (16%) and 35 eyes (35%) in the non-PM and PM groups, respectively (*P* = 0.095). In both non-PM and PM groups, the annual number of injections decreased, and almost no additional injections (mean number of injections, 0.2) were reached in the non-PM group for the 4th and in the PM group in the 3rd year (Fig. 3). The ratio of IVR against the total number of injections did not significantly differ in the non-PM and PM groups (0.70 ± 0.45 vs. 0.59 ± 0.45, *P* = 0.18).

### Morphological analysis

In all eyes of both groups, the MNV was of type 2. The baseline MNV size was not significantly different between the non-PM and PM groups (0.42 ± 0.49 mm^2^ vs. 0.57 ± 0.65 mm^2^, *P* = 0.52). The baseline MNV location was also not significantly different between the two groups (*P* = 0.67). The baseline CRT was not different between the non-PM and PM groups (288.9 ± 107.3 μm vs. 291.1 ± 153.5 μm, *P* = 0.82); whereas the baseline CCT was higher in the non-PM group than in the PM group, without reaching statistical significance (74.2 ± 36.7 μm vs. 57.9 ± 22.3 μm, *P* = 0.06). The rates of the eyes with ERM (from 20% [20/99] to 31% [31/99]) increased in the PM group over 4 years, but not in the non-PM group (from 11% [2/19] to 16% [3/19]). Furthermore, in 16 of 19 eyes (84%) in the non-PM group, non-PM progressed to PM during the 4-year observational period.

### Correlation analysis

Using multivariable correlation analyses, only baseline logMAR BCVA value significantly correlated with 4/year logMAR BCVA value in both groups (non-PM group, *P* = 0.047, β = 0.46; PM group, *P* < 0.001, β = 0.59) (Table 2). Age, AL, MNV location, MNV size, CRT, CCT, total number of injections, and cataract surgery during the 4-year observation period did not significantly correlate with the 4-year logMAR BCVA value.

**Table 2:** Correlation between the 4-year logMAR BCVA and studied parameters in the non-PM and PM groups.

		Non-PM group (n = 19)				PM group (n = 99)			
		Univariable analysis		Multivariable analysis		Univariable analysis		Multivariable analysis	
		*P*	r	*P*	β	*P*	r	*P*	β
Baseline parameters	Sex (1, male; 2, female)	0.18	− 0.33	–	–	0.11	0.16	–	–
	Age	0.98	− 0.01	–	–	0.11	0.16	–	–
	AL	0.06	− 0.44	0.15	− 0.31	0.63	0.16	–	–
	LogMAR BCVA	0.01*	− 0.62	0.047*	0.46	< 0.001*	0.59	< 0.001*	0.59
	MNV location (1, extrafoveal; 2, juxtafoveal; 3, foveal)	0.44	0.19	–	–	0.36	0.09	–	–
	MNV size	0.34	0.23	–	–	0.001*	0.33	0.92	0.009
	CRT	0.29	0.26	–	–	0.22	0.12	–	–
	CCT	0.99	− 0.004	–	–	0.47	− 0.07	–	–
	ERM (0, absent; 1, present)	0.03*	− 0.49	0.22	− 0.27	0.80	− 0.03	–	–
	LMH (0, absent; 1, present)	–	–	–	–	–	–	–	–
	Lens status (1, phakia; 2, pseudophakia or aphakia)	0.20	− 0.31	–	–	0.91	− 0.01	–	–
Treatment parameters	The number of injections	0.10	0.39	–	–	0.87	− 0.02	–	–
	Cataract surgery during the 4-year observation period	0.87	− 0.04	–	–	0.42	− 0.08	–	–

PM = pathologic myopia; logMAR BCVA = logarithm of the minimal angle of resolution best-corrected visual acuity; MNV = macular neovascularization; CRT = central retinal thickness; CCT = central choroidal thickness; ERM = epiretinal membrane; LMH = lamellar macular hole.

*Statistically significant (*P* < 0.05).

## Discussion

The present study elucidated the outcomes of anti-VEGF therapy with a 1 + PRN regimen for MNV in the eyes with pathologic myopia (PM group) and in those with non-pathologic high myopia (non-PM group) over a 4-year observation period. The 4-year course of BCVA was similar in both groups; however, more injections were required in the non-PM group, despite all eyes in both groups having type 2 MNV (type 2 responds better to anti-VEGF therapy than type 1, as previously reported)^15^. Only 16% of the eyes in the non-PM group required no additional injections during the 4-year observation period, which was lower than the PM group (35%). These results suggest that more frequent and careful follow-up is required for eyes with MNV in eyes with non-pathologic high myopia compared to those with PM.

The annual number of additional injections decreased in both groups. Our 4-year findings that the levels of almost no additional injections were reached in the non-PM group in the 4th and in the PM group in the 3rd year complement the results of a previous 2-year observational study^5^. The treatment course in the eyes with non-PM or PM was different from that in the eyes with nAMD using a PRN regimen. For nAMD, the mean annual number of IVA or IVR was reported to be 2.5–3.0, and it did not decrease during the 7-year observation period^12^. Considering the required injection number, the MNV activity in the eyes with highly myopic non-PM would range between that of the eyes with PM and that of the eyes with nAMD. We consider that the activity would be associated with the amount of blood supply from the choroid; therefore, MNV in the non-PM eyes, with thicker choroid and probably higher blood supply to MNV, would have had higher activity and required more injections than in the eyes with PM.

The 4-year BCVA significantly correlated with the baseline BCVA in multivariable analyses in both groups, which was consistent with findings of a 10-year observational study on myopic MNV with PM and a 7-year observational study on nAMD^3,12^. To maintain long-term BCVA after anti-VEGF therapy, early detection and subsequent early treatment are also important for non-PM and PM. Consistent with the findings of a 2-year observational study^13^, MNV size correlated with the 4-year BCVA value in the univariable analysis but not in the multivariable analysis. Contrary to the results of another 2-year observational report^14^, MNV locations did not correlate with the 4-year BCVA in either group, which was unexpected. The main differences between this previous study and ours were the use of bevacizumab, the exclusion of extrafoveal MNV, and a shorter observation period. Further research regarding this difference is required.

In 84% of the eyes in the non-PM group, non-PM changed to PM during the 4-year observational period, which might have induced a relatively rapid decrease of BCVA observed 1 year after the initial treatment (Fig. 2). Whether anti-VEGF therapy, MNV, or others induced the progression of the META-PM category was unclear. Comparing the category progression rate of 60% in the eyes with PM during the 10-year observational period of the previous study^3^, 84% in the present study is high; however, the rate was unclear after 4 years, and the sample size was small in the present study. A large-sample-sized and long-term observational study of the eyes with non-PM is desired.

Despite its strengths of a relatively long-term follow-up and a large sample size, the present study has several limitations. First, the retrospective study design might have led to selection bias; however, we included consecutive eyes that met the criteria during the study period to eliminate such bias as much as possible. Second, this study might have included MNV secondary to punctate inner choroiditis (PIC). Differentiating between myopic MNV and MNV secondary to PIC was reported to be challenging^16^. Most previous studies on highly myopic MNV would include eyes with PIC. The method to completely distinguish highly myopic MNV from PIC is desired. Third, IVR and IVA were mixed. However, a previous report showed that 2-year BCVA (*P* = 0.6), CRT (*P* = 0.9), and number of injections (*P* = 0.6) were similar between IVR and IVA^17^. Furthermore, the ratio of IVR against the total number of injections did not significantly differ between the two groups.

In conclusion, over the 4-year observation period, the BCVA course after anti-VEGF therapy for myopic MNV was similar in the eyes with non-PM and those with PM. However, more additional injections in a PRN regimen were required in the eyes with non-PM compared to those with PM. Thus, more frequent and careful follow-up is required for the eyes with non-PM compared to those with PM to maintain long-term BCVA.

## Data Availability

All data generated or analyzed during this study are included in this published article.
